# Micro Non-Destructive Testing and Evaluation

**DOI:** 10.3390/ma15175923

**Published:** 2022-08-27

**Authors:** Giovanni Bruno

**Affiliations:** BAM, Bundesanstalt für Materialforschung und -prüfung, Unter den Eichen 87, 12205 Berlin, Germany; giovanni.bruno@bam.de

## 1. Foreword

What is meant by ‘Micro Non-Destructive Testing and Evaluation’? This was the central subject of debate in this Special Issue.

At present, sub-millimeter-size components or even assemblies are pervading the industrial and scientific world. Classic examples are electronic devices and watches (as well as parts thereof), but recent examples encompass additively manufactured lattice structures, stents, or other microparts. Moreover, most assemblies contain micro-components. Testing such components or their miniaturized parts would fit well within the topic of micro non-destructive testing and evaluation.

In all cases, performance and integrity testing, quality control, and dimensional tolerances need to be measured at the sub-millimeter level (ideally with a spatial resolution of about a micron); most of the time, such features and components are embedded in much larger assemblies, which also need to be taken into account. The solution to this dilemma (i.e. measuring large parts with high resolution) depends on the part and on the problem under consideration.

Another possible definition of micro non-destructive testing and evaluation can relate to the characterization of micro-features (e.g., the microstructure) in much larger specimens, such as damage in concrete cores or porosity in additively manufactured components. A further aspect is the use of microscopic probes to evaluate macroscopic properties. This is the case, for instance but not at all exclusively, in the use of diffraction techniques to determine macroscopic stress.

The splits between testing and characterization at the micro-level (or of micro parts) from one side and handling of macroscopic assemblies on the other represent a great challenge for many fields of materials characterization. On top of that, including the use of microscopic methods to test integrity would add a further level of complexity.

Imaging, mechanical testing, non-destructive testing, measurement of properties, structural health monitoring, and dimensional metrology all need to be re-defined if we want to cope with the multi-faceted topic of micro non-destructive testing and evaluation.

The challenge has already been accepted by the scientific and engineering communities for a while but is still far from being universally tackled. This Special Issue yields an interesting answer to the questions posed above. It presents the progress made and the different aspects of the challenge as well as at indicates the paths for the future of NDT&E.

## 2. Introduction

With the increasing miniaturization of components, performance assessment, quality control, and structural health monitoring have expanded their toolbox of experimental techniques. Classically, non-destructive testing and evaluation (NDT&E) has included macroscopic probes such as radar, X-ray radiography, and ultrasound for structures or large components. Recently, other tools have been used to cope with the challenge of miniaturization. Such tools include not only spatially or temporally resolved techniques such as synchrotron radiation imaging but also investigation techniques, which in the past belonged more to the realm of materials science than to engineering (e.g., diffraction and laser-induced breakdown spectroscopy). However, such ‘new’ tools can be and have also been applied to investigate large components: X-ray and neutron diffraction are currently used to determine the residual stress in safety-relevant components (nuclear industry and additive manufacturing) [1,2], and X-ray computed tomography is used to investigate the degradation of concrete cores [3,4]. The meaning of micro-NDT&E (μ-NDT&E) methods has, therefore, been extended from the use of NDT&E techniques on microscopic components to the use of microscopic techniques and to macroscopic components.

## 3. Summary of the Special Issue

This Special Issue shows that X-ray computed tomography is becoming a major tool for μ-NDT&E, being used for small and large components [3,5,6], and for sensitive materials [7]. Indeed, new methods are also being developed [6,7,8]. At the same time, optical methods are being perfected to tackle challenging problems at the micro and macro scales [9,10]. Moreover, magnetic methods are still very powerful for detecting defects in several materials and components [11,12,13,14], especially when talking about large components. Indeed, such methods are being further developed and extended to new materials such as concrete [15] and to applications such as residual stress determination [16].

From the materials point of view, it is clear that concrete plays an eminent role in the field of NDT&E. Its eternal youth and wide application fields render it always useful, so that new kinds of investigations are paralleled with new compositions and materials designs [17]. However, classic materials such as steels [13,18], novel metallic biomaterials [19], and additively manufactured metallic alloys and structures [5,10] are also at the top of the agenda.

## 4. Conclusions

From the discussion above, we conclude that, in general, NDT&E methods are of primary importance in the design, performance assessment, quality control, and structural health monitoring of materials and components (metallic or ceramic/cementitious). One could summarize the meaning of the contributions to this Special Issue in a nutshell by stating that, currently, there is no separation between micro-NDT&E and NDT&E, since the latter field already includes the first and the applications of NDT&E methods to miniaturized materials or to the microscopic scale (materials science and characterization) has already been happening for some time.

## Data Availability

Not applicable.
